# HER2 Aberrations as a Novel Marker in Advanced Biliary Tract Cancer

**DOI:** 10.3389/fonc.2022.834104

**Published:** 2022-02-14

**Authors:** Hongsik Kim, Ryul Kim, Hye Ryeon Kim, Hyunji Jo, Hana Kim, Sang Yun Ha, Joon Oh Park, Young Suk Park, Seung Tae Kim

**Affiliations:** ^1^ Division of Hematology-Oncology, Department of Medicine, Samsung Medical Center, Sungkyunkwan University School of Medicine, Seoul, South Korea; ^2^ Division of Hematology-Oncology, Department of Internal Medicine, Chungbuk National University Hospital, Cheongju, South Korea; ^3^ Department of Pathology, Samsung Medical Center, Sungkyunkwan University School of Medicine, Seoul, South Korea

**Keywords:** HER2, ERBB2, biliary tract cancer, next-generation sequencing, chemotherapy

## Abstract

HER2 aberrations have been reported as a novel biomarker in HER2-directed therapy or as a prognostic marker in various tumor types. However, in advanced biliary tract cancer (BTC), there have been few studies regarding HER2 aberrations as a biomarker. We analyzed 121 advanced BTC patients who had been treated with Gemcitabine/Cisplatin (GP) as a 1st line therapy between November 2019 and April 2021. Next-generation sequencing (NGS), namely, HER2 aberrations was performed in all patients. The TruSight™ Oncology 500 assay from Illumina was used for the NGS panel. Among 121 patients with advanced BTC, HER2 aberrations were observed in 18 patients (14.9%). For subtypes of HER2 aberrations, point mutation was observed in 5 patients (27.8%), gene amplification in 11 patients (61.1%), and both point mutation and gene amplification in 2 patients (11.1%). The frequency of HER2 aberrations was significantly different according to the primary tumor (p = 0.009). In gallbladder cancer, HER2 aberrations were observed at a relatively high frequency (36.4%). The tumor response to GP did not differ between patients with and without HER2 aberrations (33.3%, vs. 26.2%, respectively, p = 0.571). The median progression-free survival (PFS) to GP was 4.7 months (95% CI, 4.0 to 5.5 months) in patients with HER2 aberrations and 7.0 months (95% CI, 5.2 to 8.8 months) without HER2 aberrations (p = 0.776). The median overall survival (OS) was not reached and not reached in patients with and without HER2 aberrations (p = 0.739), respectively. The univariate analysis for PFS to GP and OS showed that HER2 aberrations were not an independent factor for survival. This study showed that the HER2 aberrations were observed in 14.9% of advanced BTC and were not an independent biomarker for survival.

## Introduction

Biliary tract cancers (BTCs) are rare, aggressive, and heterogeneous malignancies (1, 2). Most patients present with advanced disease at the time of diagnosis. Palliative chemotherapy is the only treatment option for advanced BTC, and the gemcitabine plus cisplatin (GP) has been the standard of treatment as 1st line chemotherapy. However, the prognosis for these patients is poor, and median overall survival (OS) is less than one year with palliative chemotherapy (3–5).

Human epidermal growth factor receptor 2 (HER2) is associated with tumor proliferation by downstream signaling activation and is among the most investigated biomarker in various tumor types, namely, breast and gastric cancers (6, 7). HER2 aberrations play a role as predictive and prognostic biomarkers in various tumor types (8–11). Several studies also reported that the HER2 pathway could have a role in the development and growth of BTC (12–15) and HER2 overexpression and amplification were reported approximately 4–6% of BTC, 1–4% of intrahepatic cholangiocarcinoma, 4–9% of extrahepatic cholangiocarcinoma, and 9–14% of gallbladder cancer (11, 15, 16). Also, HER2-directed therapy has been developed in advanced BTCs (17–22). Several previous studies have assessed HER2 overexpression and amplification by immunohistochemistry (IHC) and focused only on HER2 directed therapy based on the results of IHC. However, most studies have focused only on the overexpression and/or amplification of HER2.

Recently, advances in whole-exome sequencing (WES) and next-generation sequencing (NGS) of multiple genes have defined the tumor biology of BTCs (23–25). Also, in previous studies, HER2 aberrations by NGS highly correlated with HER2 overexpression by IHC/FISH in various solid tumors (26–28). Currently, several clinical trials are evaluating the HER2-directed therapy based on HER2 aberrations detected by NGS in advanced BTCs (29, 30). However, in the era of NGS, there have been few reports on the role of HER2 aberrations, namely, gene mutation, gene amplification, and overexpression as a biomarker in advanced BTCs (31–34) and the role of HER2 aberrations to cytotoxic chemotherapy has not been evaluated yet. Therefore, we intended to explore the prevalence of HER2 aberrations using NGS in advanced BTCs and evaluate the role of HER2 aberrations as both a predictive factor for GP and a prognostic factor.

## Materials and Methods

### Patients

We analyzed 121 advanced BTC patients who received gemcitabine and cisplatin (GP) as the 1st line treatment at the Samsung Medical Center, Korea, between November 2019 and April 2021. Molecular profiles, namely, HER2 aberrations, were available for all patients through NGS using the TruSight™ Oncology 500 assay (Illumina Inc., San Diego, CA, USA). The baseline clinicopathologic characteristics were collected for patients. The Institutional Review Board (IRB No. 2021-07-110) at the Samsung Medical Center approved this study and this retrospective analysis waived individual consent. 

### TruSight™ Oncology 500 Assay

The tumor samples were obtained at the time of diagnosis in advanced or metastatic BTCs and used formalin-fixed paraffin-embedded (FFPE) material. For DNA library preparation and enrichment, the TruSight™ Oncology 500 Kit was used following the manufacturer’s instructions. Post-enriched libraries were quantified, pooled, and sequenced on a NextSeq 500. The quality of the NextSeq 500 sequencing runs was assessed using the Illumina Sequencing Analysis Viewer. Sequencing data were analyzed with the TruSight Oncology 500 Local App Version 1.3.0.39. The TruSight™ Oncology 500 is a comprehensive tumor profiling assay and biomarkers, namely, single nucleotide variants (SNVs), copy number variants (CNVs), indels, fusions, and splice variants.

### Treatment Outcomes

All 121 patients were evaluated for clinical outcomes of objective response rate (ORR), disease control rate (DCR), and progression-free survival (PFS) to gemcitabine and cisplatin as the 1st line treatment according to the Response Evaluation Criteria in Solid Tumors (RECIST) version 1.1 through computed tomography (CT). Also, overall survival (OS) was analyzed. PFS was defined as the time from the start of GP until the date of disease progression or death from any cause. OS was defined as the time from the start of GP until death from any cause. According to the RECIST, ORR was defined as the proportion of patients with a complete response (CR) or partial response (PR) to treatment and DCR was defined as the proportion of patients with a complete response (CR), partial response (PR) or stable disease (SD) to treatment.

### Statistics

The cut-off date for data collection was April 30, 2021. Descriptive statistics were used to summarize patient and tumor characteristics and treatment history and were reported as proportions and medians. Data are presented as the number (%) for categorical variables. Correlations between HER2 aberrations and clinicopathologic features were analyzed by t-test or Fisher exact test. Survival analyses were performed using the Kaplan–Meier method, and differences were analyzed by log-rank test. Hazard ratios and corresponding 95% confidence intervals were calculated using the Cox proportional hazards model. Univariate analysis of predictive and prognostic factors was performed using Cox proportional hazards models for PFS and OS. IBM SPSS Statistics 25 was used for statistical analysis.

## Results

### Patient Characteristics and HER2 Aberrations

All 121 patients were analyzed in this study. Of these, 18 (14.9%) had tumors with HER2 aberrations. HER2 aberrations were found in 5.8% (3/52) of intrahepatic cholangiocarcinoma patients, 13.9% (5/36) of extrahepatic cholangiocarcinoma patients, 36.4% (8/22) of gallbladder cancer patients, and 18.2% (2/11) of ampulla of Vater cancer patients. For subtypes of HER2 aberrations, point mutation was observed in 5 patients (27.8%), gene amplification in 11 patients (61.1%), and both point mutation and gene amplification in 2 patients (11.1%). **Table 1** presents the clinical characteristics between patients with and without HER2 aberrations. HER2 aberrations were not significantly correlated with any clinical baseline characteristics except the location of the primary tumor (p = 0.009).

**Table 1:** Baseline patient characteristics.

	HER2 (−) (n = 103)	HER2 (+) (n = 18)	p-value
Median age (range), years	64 (47–79)	67 (33–82)	0.580
Age 65≥ years, n (%)	58 (56.3%)	8 (44.4%)	0.499
Sex, n (%)			0.556
Male	68 (66.0%)	10 (55.6%)	
Female	35 (34.0%)	8 (44.4%)	
Tumor site, n (%)			0.009
Intrahepatic	49 (47.6%)	3 (16.7%)	
Extrahepatic	31 (30.1%)	5 (27.8%)	
Gallbladder	14 (13.6%)	8 (44.4%)	
Ampulla of Vater	9 (8.7%)	2 (11.1%)	
Grade of differentiation, n (%)			0.838
Poorly	29 (28.2%)	4 (22.2%)	
Well/Moderate	67 (65.0%)	13 (72.2%)	
Unknown	7 (6.8%)	1 (5.6%)	
Disease stage, n (%)			0.626
Metastasis	77 (74.8%)	15 (83.3%)	
Locally advanced	26 (25.2%)	3 (16.7%)	
No. of metastatic sites, n (%)			0.928
≤2	90 (87.4%)	15 (83.3%)	
2<	13 (12.6%)	3 (16.7%)	
Metastatic sites, n (%)			
Abdominal lymph node (M1)	45 (43.7%)	10 (55.6%)	
Liver	41 (39.8%)	8 (44.4%)	
Peritoneum	20 (19.4%)	4 (22.2%)	
Lung	6 (5.8%)	1 (5.6%)	
Bone	4 (3.9%)	1 (5.6%)	
Others	17 (16.5%)	6 (33.3%)	

HER2, human epidermal growth factor receptor 2.

### Association Between the Status of HER2 Aberrations and the Efficacy of Gemcitabine Plus Cisplatin

We compared the tumor response, PFS, and OS to GP according to the status of HER2 aberrations. The ORR and DCR to GP were 33.3% (6/18, 95% CI 13.3–59.0) and 77.8% (14/18, 95% CI 52.3–93.6), respectively, in patients with HER2 aberrations and 26.2% (27/103, 95% CI 18.0–35.8) and 73.8% (76/103, 95% CI 64.2–82.0), respectively, in patients without HER2 aberrations. The ORR and DCR according to the status of HER2 aberrations were no significant differences (p = 0.571 and p = 1.000, respectively) (**Table 2**).

**Table 2:** Objective response rate to chemotherapy.

	HER2 aberrations (−) (n = 103)	HER2 aberrations (+) (n = 18)	p-value
Complete response	2 (1.9%)	1 (5.6%)	
Partial response	25 (24.3%)	5 (27.8%)	
Stable disease	49 (47.6%)	8 (44.4%)	
Progressive disease	14 (13.6%)	1 (5.6%)	
Not evaluable	13 (12.6%)	3 (16.7%)	
Objective response rate	27 (26.2%)	6 (33.3%)	0.571
Disease control rate	76 (73.8%)	14 (77.8%)	1.000

HER2, human epidermal growth factor receptor 2.

**Figure 1:**
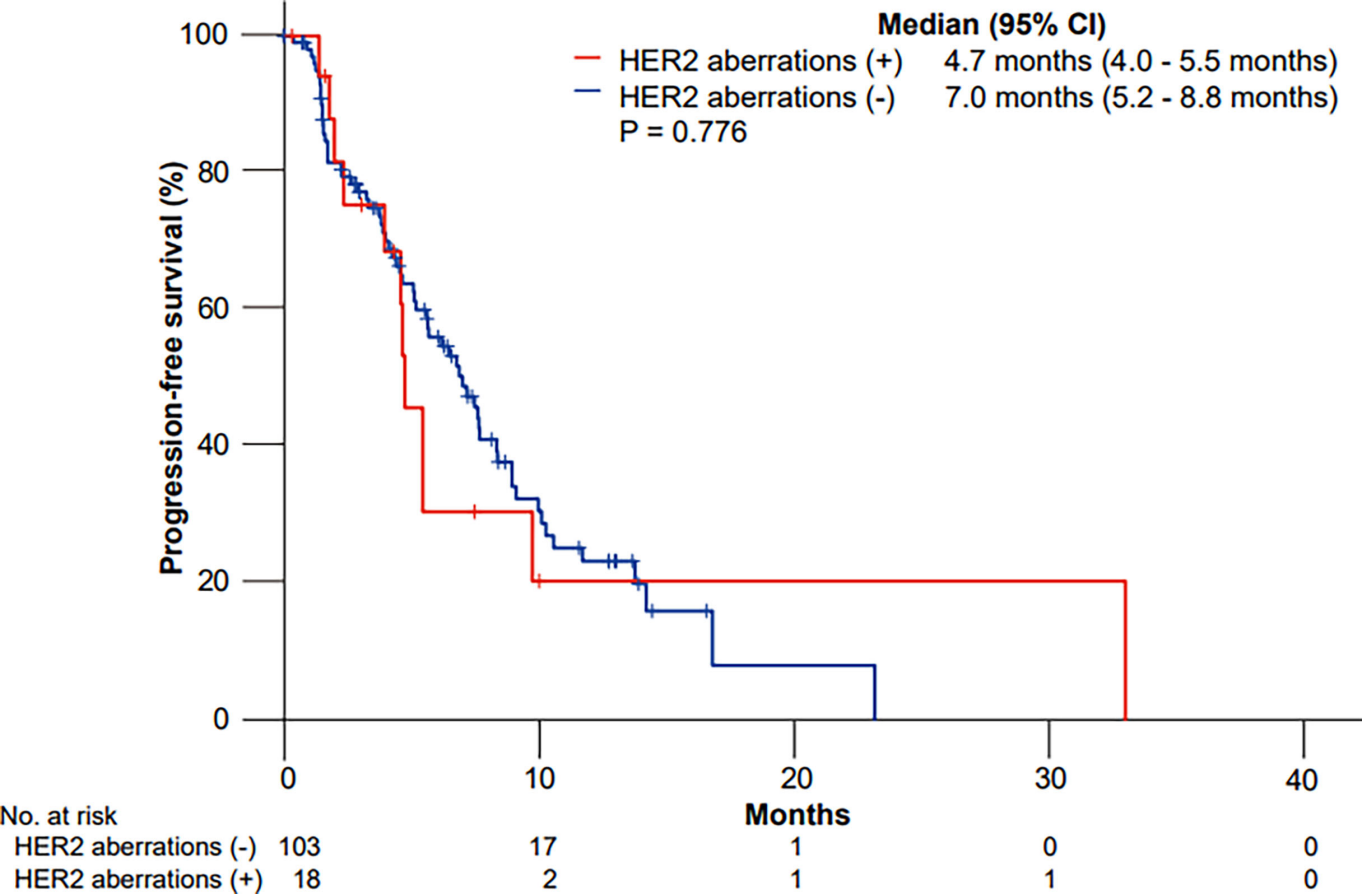
Kaplan–Meier curves of progression-free survival (PFS) to gemcitabine plus cisplatin according to HER2 aberrations.

The median PFS to GP values was 4.7 months (95% CI, 4.0 to 5.5 months) and 7.0 months (95% CI, 5.2 to 8.8 months) in patients with and without HER2 aberrations, respectively (p = 0.776) (**Figure 1**).

**Figure 2:**
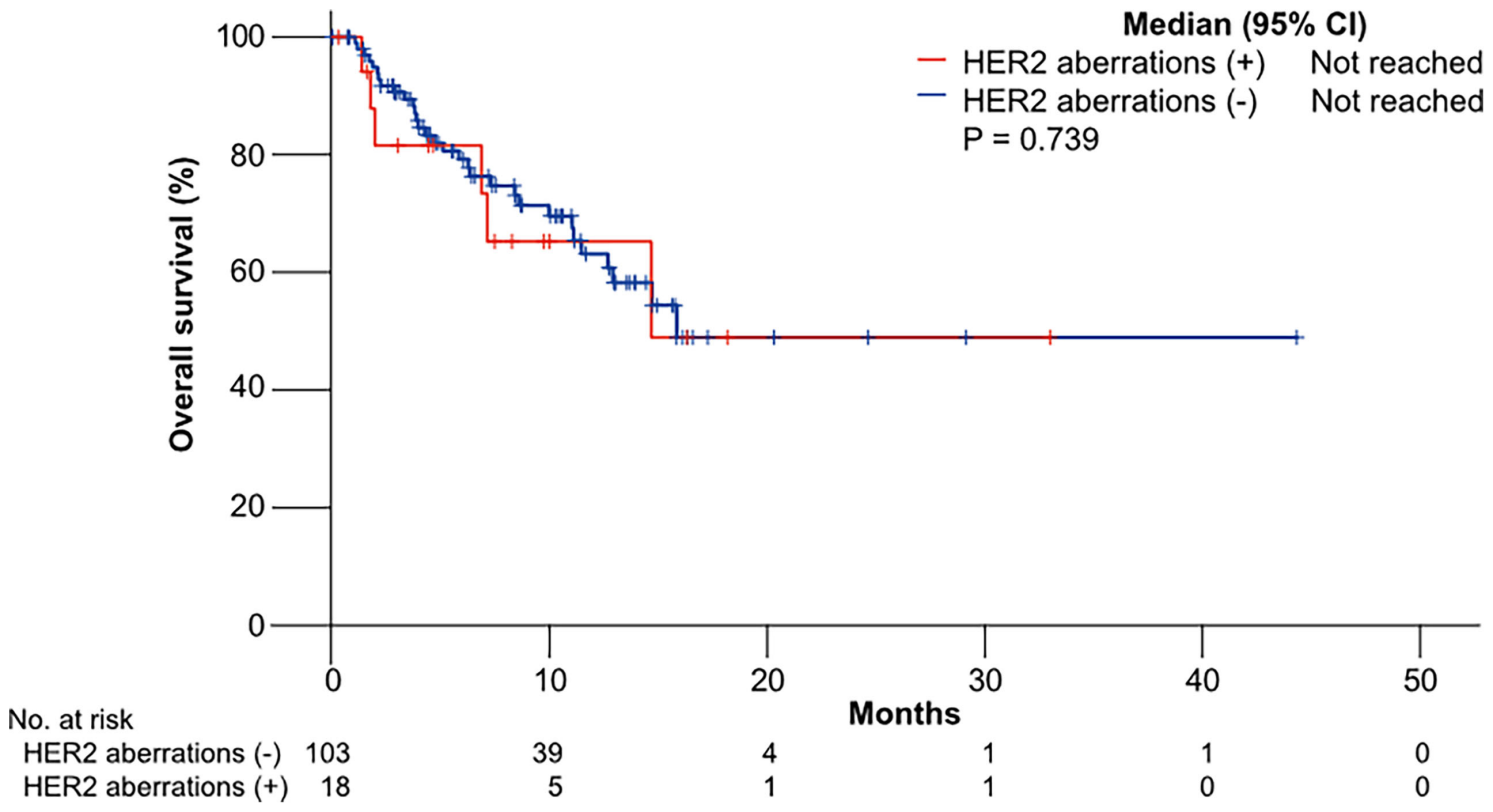
Kaplan–Meier curves of overall survival (OS) to gemcitabine plus cisplatin according to HER2 aberrations.

The median OS was not reached in patients with HER2 aberrations and not reached in patients without HER2 aberrations (p = 0.739) (**Figure 2**).

### HER2 Aberrations—Univariate Analysis for Survivals

We conducted univariate analyses for PFS to GP and OS to evaluate the role of HER2 aberrations as an independent biomarker (**Table 3**). Univariate analysis for PFS to GP showed that grade of differentiation (poorly differentiated vs. well/moderate differentiated), disease stage (metastasis vs. locally advanced), and the number of metastatic sites (≤2 vs. 2<) were significant independent factors; however, HER2 aberrations were not.

**Table 3:** Univariate analysis of progression-free survival and overall survival after gemcitabine/cisplatin.

	Progression-free survival		Overall survival	
	HR (95% CI)	p-value	HR (95% CI)	p-value
**Age**		0.288		0.727
<65 years	1		1	
≥65 years	1.29 (0.81–2.07)		1.12 (0.59–2.15)	
**Sex**		0.139		0.010
Male	1		1	
Female	0.69 (0.43–1.12)		0.35 (0.16–0.78)	
**Tumor site**				
Intrahepatic	1	0.395	1	0.097
Extrahepatic	0.89 (0.51–1.56)	0.685	0.75 (0.36–1.57)	0.444
Gallbladder	0.82 (0.44–1.54)	0.537	0.54 (0.20–1.44)	0.219
Ampulla of Vater	0.46 (0.19–1.12)	0.087	0.07 (0.02–1.20)	0.074
**Grade of differentiation**		0.014		0.012
Poorly	1		1	
Well/Moderate	0.53 (0.32–0.88)		0.42 (0.22–0.83)	
**Disease stage**		0.006		0.142
Metastasis	1		1	
Locally advanced	0.27 (0.11–0.69)		0.34 (0.08–1.43)	
**No. of metastatic sites**		0.004		0.001
≥2	1		1	
<2	2.14 (1.27–3.62)		3.63 (1.87–7.03)	
**HER2 aberrations**		0.771		0.739
Negative	1		1	
Positive	1.10 (0.58–2.11)		1.16 (0.48–2.79)	

HER2, human epidermal growth factor receptor 2; HR, hazard ratio; CI, confidence interval.

Also, HER2 aberrations were not an independent factor in univariate analysis for OS. We additionally conducted survival analyses for PFS and OS according to HER2 amplification. HER2 amplification also was not an independent factor in univariate analysis for PFS (p = 0.322) and OS (p = 0.168).

## Discussion

In our study, we identified that the prevalence of HER2 aberrations and the frequency of HER2 aberrations were significantly different according to the primary tumor, which was consistent with previous studies (16, 23, 35). Between the status of HER2 aberrations and treatment outcomes to GP, including ORR, DCR, and PFS were no significant differences. Also, HER2 aberrations were not an independent biomarker for PFS to GP and OS in univariate analysis. Because most clinical trials of HER2 targeted therapy included HER2 amplified BTCs, we additionally evaluate the role of HER2 amplification as a prognostic biomarker. However, HER2 amplification was not an independent biomarker for OS.

Recently, one study described genomic characteristics focusing on the ERBB/EGFR pathway in BTC using NGS. The prevalence of HER2 aberrations by NGS was 13.9% in 1,863 BTC patients from Western countries; of these, 6.2% were point mutation, 6.8% were amplification, and 0.9% were both point mutation and amplification, which was consistent with our results (14.9% in 121 patients) (16). However, that study did not evaluate the role of HER2 aberrations as predictive and prognostic biomarkers.

The role of HER2 aberrations as a predictive and prognostic biomarker in advanced BTC to palliative chemotherapy has not previously been elucidated. One study reported that HER2 expression by IHC represented an independent poor prognostic factor in patients with BTC treated with curative surgery (31). That study evaluated the relationship between HER2 expression by IHC and survival in 100 patients with radically resected BTC. However, there was a lack of standardized criteria of HER2 assessment in BTC, and the patient groups appeared unbalanced according to HER2 status. Meanwhile, another study reported that HER2 overexpression by IHC was not a significant difference in survival rates in BTC patients with curative surgery (36). Similarly, our results for the prognostic role of HER2 aberrations were inconsistent and inconclusive.

Several clinical trials with novel tyrosine kinase inhibitors (such as lapatinib or erlotinib) (37, 38) and monoclonal antibodies (such as cetuximab or panitumumab) (39, 40) targeting the HER pathway have been developed in HER2 overexpressing BTC and brought disappointing results. Recently, in the MyPathway HER2 basket study, combined therapy with pertuzumab plus trastuzumab resulted in an ORR of 9 of 39 patients (23%) with metastatic BTCs with HER2 amplification/overexpression, and the median OS was 10.9 months (21). Also, promising results from a phase 1 study were reported from new drugs, such as the novel HER2-targeted antibody-drug conjugate trastuzumab deruxtecan (T-Dxd) (41), the anti-HER2 antibody margetuximab (MGAH22) (42), and the bispecific HER2-targeted antibody zanidatamab (ZW25) (43). Accumulating data provide the potential benefit from HER2-targeted therapies in HER2-positive BTCs.

Recently, neoadjuvant chemotherapy is considered a promising option for patients with curative intended surgery and a few clinical studies of neoadjuvant chemotherapy have been reported (44). Although our focused only on the role of HER2 aberrations as a novel biomarker by NGS in advanced BTC to palliative chemotherapy, further studies are needed to evaluate the aberrations of HER2 as a biomarker in the setting of neoadjuvant chemotherapy.

Although the role of HER2 in BTC patients is inconsistent and inconclusive, previous studies have reported HER2 overexpression by IHC as a predictive and prognostic biomarker. Recently, in the era of NGS, clinical trials with HER2 targeting therapy included HER2 aberrations by NGS. Therefore, we reviewed the prevalence of HER2 aberrations using NGS in advanced BTCs and a new perspective which was a prognostic and predictive role of HER2 aberrations by NGS in advanced BTCs.

To the best of our knowledge, ours is the first study evaluating relationships between HER2 aberrations and GP, which is the current standard chemotherapy. We found that HER2 aberrations identified through NGS did not have a predictive or prognostic role on the standard first-line chemotherapy in advanced BTC.

This study has limitations. First, this study had a small sample size, was retrospective in nature, and utilized a heterogeneous population, which may lead to bias. However, the biliary tract cancers were a rare orphan disease. Especially, the acquisition of tumor-sample in biliary tract cancers is very difficult to work. This study tried to conduct a molecular study in biliary tract cancer. Second, only Asian patients with BTC were analyzed in the study, limiting the generalizability because of differences in molecular profiles and clinical features between Western and Eastern patients with BTC. Third, the study included various types of HER2 aberrations, making it difficult to draw definite conclusions. Therefore, findings for the HER2 aberrations as a novel biomarker in this study should be interpreted with caution. Further prospective clinical trials are required to determine whether HER2 aberrations could be a novel predictive or prognostic biomarker in BTC.

In conclusion, this retrospective study evaluated the prevalence of HER2 aberrations in BTC and the relationship between HER2 aberrations and clinical outcomes after cytotoxic chemotherapy. Our results suggest that HER2 aberrations in advanced BTC did not have a prognostic or predictive biomarker in the first line standard cytotoxic chemotherapy (GP).

## Data Availability Statement

The datasets presented in this article are not readily available because of the privacy restrictions at Samsung Medical Centre. Requests to access the datasets should be directed to shty1@skku.edu.

## Ethics Statement

The studies involving human participants were reviewed and approved by the Institutional Review Board (IRB no. 2021-07-110) of Samsung Medical Centre. 

## Author Contributions

Conception and design: HoK, SK. Provision of study materials or patients: HoK, SH, JP, YP, SK. Collection and assembly of data: HoK, RK, HRK, HJ, HaK, SK. Data analysis and interpretation: HoK, SK. Manuscript writing: HoK, SK. All authors listed have made a substantial, direct, and intellectual contribution to the work and approved it for publication.

## Conflict of Interest

The authors declare that the research was conducted in the absence of any commercial or financial relationships that could be construed as a potential conflict of interest.

## Publisher’s Note

All claims expressed in this article are solely those of the authors and do not necessarily represent those of their affiliated organizations, or those of the publisher, the editors and the reviewers. Any product that may be evaluated in this article, or claim that may be made by its manufacturer, is not guaranteed or endorsed by the publisher.
